# Thoracic endovascular aortic repair and optimal medical treatment for acute type B penetrating aortic ulcer associated with intramural hematoma

**DOI:** 10.1097/MD.0000000000031301

**Published:** 2022-11-11

**Authors:** Xiuchun Xu, Feng Lu, Li Li

**Affiliations:** a Department of General Surgery, Binhai People’s Hospital, Yancheng, Jiangsu Province, People’s Republic of China; b Department of Clinical Laboratory, Binhai People’s Hospital, Yancheng, Jiangsu Province, People’s Republic of China.

**Keywords:** intramural hematoma, penetrating aortic ulcer, thoracic endovascular aortic repair

## Abstract

To compare the safety and efficacy of thoracic endovascular aortic repair (TEVAR) and optimal medical treatment (OMT) for type B penetrating aortic ulcer (PAU) associated with intramural hematoma (IMH). From January 2015 to December 2018, 68 consecutive patients with acute type B PAU associated with IMH were enrolled in the study. TEVAR was performed following initially OMT in 30 patients (group A), and OMT was performed in 38 patients (group B). Primary outcome was aortic-related mortality. Secondary outcomes included all-cause mortality, aortic-related adverse events, and complete aortic remodeling. There was no significant difference in the baseline characteristics of patients among the 2 groups except for the depth of PAU and the thickness of IMH. Patients in group B had a significant higher risk of aortic-related mortality (13.3% vs 0%, *P* = .045), as the same to aortic-related adverse events during follow-up. Compared to OMT, TEVAR contributed to the favorable aortic remodeling more significantly during the mid-term follow-up (85.7% vs 18.2%, *P* < .001). Comparing with optimal medical repair, TEVAR for patients with PAU associated with IMH could promote the favorable aortic remolding more significantly and result in lower aortic-related mortality during mid-term follow-up. It should be considered as the first-line therapeutic option when intervention is required.

## 1. Introduction

Penetrating aortic ulcer (PAU), belonging to the spectrum of acute aortic syndrome, was described as a pathologic disorder with localized disruption of the intima and media and was first reported by Stanson and colleagues in 1986.^[1]^ Urgent open surgery serves as the routine approach to prevent PAU rupture. Thoracic endovascular aortic repair (TEVAR) has been considered to be an alternative to open surgery with lower rate of peri-procedural mortalities and complications.^[2]^

According to the previous studies, TEVAR was only considered for symptomatic patients or those with increase of the total aortic diameter at the level of the PAU, and the outcomes of emergent procedures remained significantly worse than elective intervention.^[3,4]^ For patient with uncomplicated type B PAU, medical management acts as the first line treatment and seems to provide acceptable results. However, type B PAU with high-risk features, such as the presence of a pleura effusion and intramural hematoma (IMH), seems to be at high risk of progression. Approximately 40% to 50% patients with acute type B PAU progress to aortic dissections or rupture of the aorta.^[5]^

Nowadays, no generally accepted therapeutic option has been established for this subgroup of patient. The prognosis and management of type B PAU associated with IMH remains controversial. Therefore, we aimed to compare the outcomes of TEVAR and optimal medical treatment (OMT) for the management of type B PAU associated with IMH and present our experience.

## 2. Methods

### 2.1. Patients

The retrospective study was approved by the local ethics committee, and all patients provided written informed consent. From January 2015 to December 2018, 145 patients diagnosed with PAU in our hospital were analyzed, and all cases were identified retrospectively through a review of the hospital’s documents and imaging system. Twenty patients with type A PAU and 57 patients without IMH were excluded. Type B PAU associated with IMH was confirmed in 68 patients. Baseline characteristics were collected from medical records and imaging system. Patients with the following characteristics were indicated for TEVAR: refractory hypertension and pain, rupture, peri-aortic hematoma, pleural effusion, imaging progression, and the presence of a saccular aneurysm of any size in asymptomatic patient. The primary technical success is defined as complete exclusion of the PAU in the absence of type Ia and II endoleak. Primary outcome was aortic-related mortality. Secondary outcomes included all-cause mortality, aortic-related adverse events, and incidence of complete aortic remodeling. Imaging follow-up with computed tomography angiography (CTA) was arranged for all patients before discharge, at the intervals of 3, 9 months, and then annually.

### 2.2. Pre-procedural evaluation

CTA was performed for all patients before the procedure. With the assistance of EndoSize (Therenva SAS, France), the diameter of proximal and distal landing zone, ulcer size and the extent and thickness of IMH were measured by 2 experts with more than 10 years of experience in TEVAR. No >10% of oversize was applied.

### 2.3. Treatment strategy

**Figure 1.**
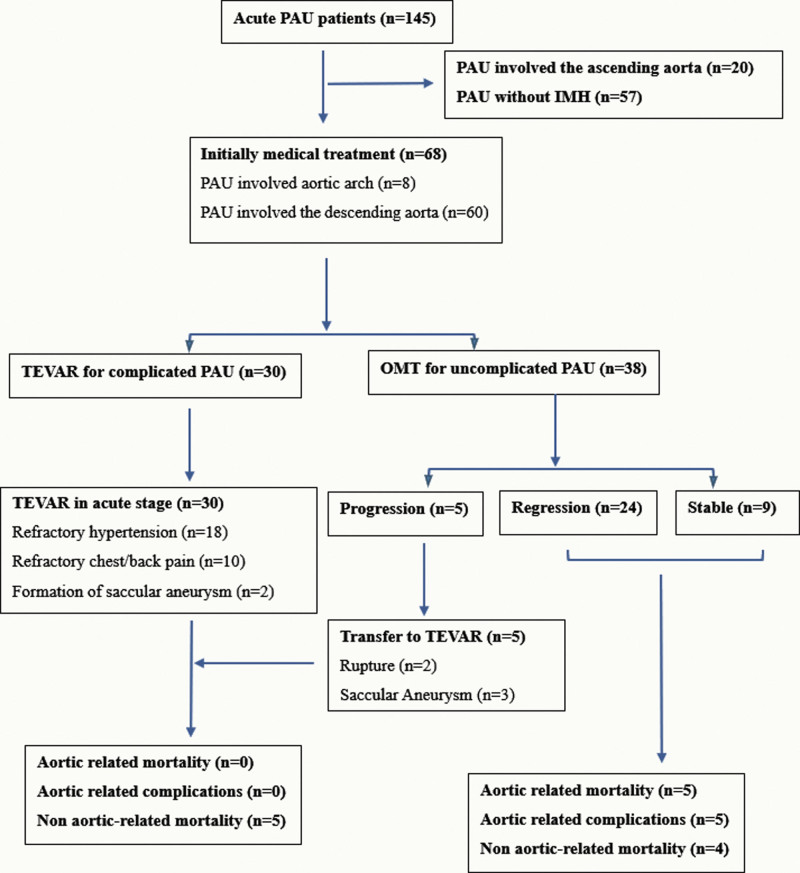
The flow chart of treatment selection.

The flow chart of treatment strategy was listed in Figure 1. Antihypertensive medical management (calcium-channel blockers, nitroglycerin, beta blockers, or combinatory treatment) was arranged immediately at the onset of the symptoms. The initial goal of medical treatment was to reduce systolic blood pressure no greater than 120 mm Hg. Analgesics were administered to relieve pain completely. TEVAR was indicated for patients with high-risk features listed above.

All TEVAR were performed in a hybrid room under general anesthesia with tracheal intubation. The left brachial artery (LBA) was obtained for deployment of a 6F sheath (Terumo corporation, Tokyo, Japan), and then a 5F pigtail catheter (Cook Medical, Bloomington) was advanced into the ascending aorta for angiography. Access into the common femoral artery (CFA) was performed with a small incision in the groin. A super stiff wire (Cook Medical, Bloomington) was advanced into the ascending aorta, and then proper deployment of the stent-graft (Life-tech Scientific, Shenzhen, China) was performed under transient apnea with the systolic blood pressure no >100 mm Hg.

Deployment of the Castor stent-grafts (Microport Medical, Shanghai, China) was performed in 6 patients, in whom PAU located ≤15 mm distal to the LSA (≥15 mm distal to the left common carotid artery) or IMH involving the ostium LSA or zone 2. Angiography and stent-graft delivery were performed according to the following standard protocol. Percutaneous retrograde brachial artery access was obtained for deployment of the LSA branch, and a CFA access was conducted for angiography. Through the sheath (Terumo corporation, Tokyo, Japan) inserted into the contralateral CFA previously, A 5F calibrated pigtail catheter (Cook Medical, Bloomington) was advanced into the ascending aorta to perform aortography. Meanwhile, the castor-branched stent-graft delivery system (Microport Medical, Shanghai, China) was advanced into the proper position in line with the super stiff wire (Cook Medical, Bloomington). The main body was deployed into the proper position and the LSA branch section was released via removal of the soft sheath. Postoperative angiography was performed to confirm the exclusion of PAU and the patency of LSA.

### 2.4. Statistical analysis

Data were reported as mean ± standard deviation and percentages. Mean values were compared using Student’s *t* test, and categorical variables were compared using the Pearson Chi-square test and Fisher’s exact test. Kaplan–Meier curves were used to determine differences in aortic-related mortality, all-cause mortality, and complete aortic remodeling. *P* value of <.05 was considered to be statistically significant. All statistical analysis was performed using the SPSS software package (IBM Corp, Armonk, NY).

## 3. Results

### 3.1. Baseline characteristics

TEVAR was performed following initially OMT in 30 patients, and OMT was administered in 38 patients. The patients’ baseline characteristics, including age, sex, history of hypertension, and presence of diabetes mellitus, renal insufficiency, or coronary artery disease are shown in Table 1. No significant difference was detected. The morphology of the PAU and IMH is also described in Table 1. The PAU was much deeper in group A than in group B (*P* < .001), and the IMH was much thicker in group A than in group B (*P* < .001). The width of PAU was not significantly different among the 2 groups (*P* = .815). There was no significant difference between the 2 groups for the extent of IMH (*P* = .703).

**Table 1:** Basic characteristics of patients.

Variables	TEVAR (n = 30)	OMT (n = 38)	*P*
Age, yr	62.27 ± 8.83	62 ± 8.96	.900
Gender, M	21	24	.554
Comorbidities, n			
Hypertension	22	25	.504
Atherosclerosis	12	14	.790
Coronary artery disease	6	7	.869
Diabetes mellitus	8	7	.416
Renal insufficiency	2	3	1
Smoking, n	20	24	.764
Location of PAU			
Zone 2, n	6	2	
Zone 3, n	24	36	.135
PAU and IHM characteristics			
PAU width, mm	16.23 ± 5.67	15.95 ± 3.62	.815
PAU depth, mm	10.97 ± 2.34	6.53 ± 2.33	<.001※
IMH thickness, mm	10.8 ± 1.61	8.53 ± 1.87	<.001※
Extent of IMH			
Confined in thoracic aorta	25	34	
Extended to abdominal aorta	5	4	.703

IMH = intramural hematoma, OMT = optimal medical treatment, PAU = penetrating aortic ulcer, TEVAR = thoracic endovascular aortic repair.

### 3.2. Peri-procedural outcomes

The peri-procedural outcomes are listed in Table 2. All procedures were performed within 14 days from onset of the symptom. With the average procedural duration of 90.53 ± 20.66 minutes, TEVAR was performed successfully in all patients. There was no incidence of paraparesis or other TEVAR-related neurologic complications. The Castor-branched stent-grafts, a kind of custom-made single-branched device, were deployed in 6 patients. Five patients had IMH extended to the abdominal aorta, and in whom an additional stent-graft was deployed for the achievement of preferable distal landing zone. One patient with slight type I endoleak was detected upon immediate angiography after deployment of the stent-graft, which disappeared upon repeated angiography about 10 minutes later. One retrograde type A dissection was detected 3 days after TEVAR, and the patient was transferred to open surgery.

**Table 2:** Procedure details of TEVAR.

Variables	TEVAR (n = 30)
Procedure duration, min	90.53 ± 20.66
Onset to TEVAR, hr	23 ± 13.03
Postoperative hospitalization, d	14.68 ± 2.20
Technique success rate	30/30
Number of stents	1.17 ± 0.38
Complication, n	
Type I endoleak, n	1
Retrograde type A aortic dissection, n	0
Stroke, n	0
30-d mortality, n	1
Type of stent-graft	
Ankura	29
Castor	6

TEVAR = thoracic endovascular aortic repair.

### 3.3. Aortic-related mortality

**Figure 2.**
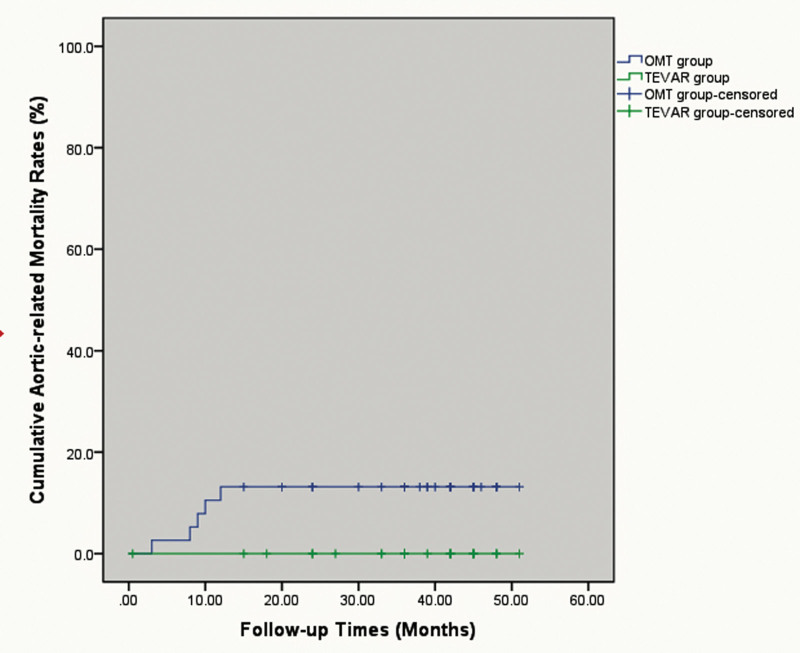
The cumulative aortic related mortality.

CTA was performed for all patients during follow-up. There was no incidence of 30-day mortality in group B, and 1 patient died within 30 days as a result of acute myocardial infarction in group A. During the mean follow-up of 36.13 ± 11.4 months in group A, no aortic-related mortality occurred, and 5 aortic-related mortalities were noted in group B with a mean follow-up of 33.76 ± 13.29 months (0% vs 13.3%, *P* = .045) (Fig. 2). Three patients died of thoracic aortic rupture, 1 patient died of abdominal aortic rupture, and 1 died of rupture of the ascending aorta. Compared with TEVAR, patients managed by OMT seemed to have a higher risk of aortic-related mortality during follow-up.

### 3.4. All-cause mortality, aortic-related adverse events, and aortic remodeling

**Figure 3.**
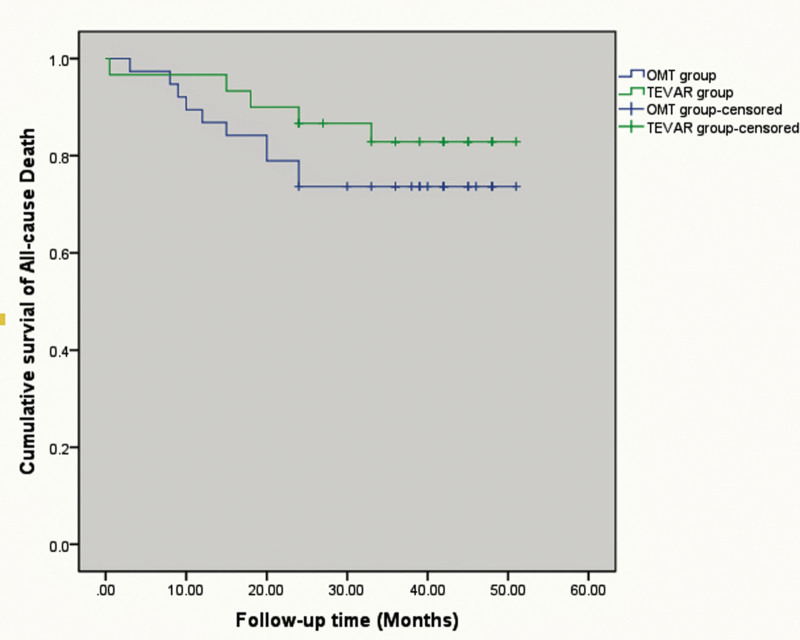
The freedom from all-cause mortality.

During follow-up, there were 4 non-aortic-related death in group B (1 died as a result of coronary artery, 1 died as a result of pulmonary failure, 1 died as a result of car accident, and 1 died with no confirmed cause). Five deaths were noted in group A. The cause included acute myocardial infarction, chronic heart failure, and lung cancer. There was also 1 death with no confirmed cause (Table 3). The causes of all mortality are listed in Table 3. There was no significant difference for all-cause mortality among the 2 groups (*P* = .327) (Fig. 3).

**Table 3:** Causes of all mortality.

Gender	Age, yr	Treatment	SG	Cause	FU, mo
M	58	OMT	-	No confirmed cause	24
M	47	OMT	-	Aortic rupture	3
F	70	OMT	-	Aortic rupture	10
F	77	OMT	-	Pulmonary failure	20
M	78	OMT	-	Aortic rupture	8
F	73	OMT	-	CAD	20
M	62	OMT	-	Retrograde TAAD	12
F	70	OMT	-	Aortic rupture	9
M	68	OMT	-	Car accident	24
M	65	TEVAR	Ankura	Acute myocardial infarction	0.5
M	70	TEVAR	Castor	Lung cancer	24
M	78	TEVAR	Ankura	Heart failure	33
F	68	TEVAR	Ankura	No confirmed cause	18
F	70	TEVAR	Ankura	CAD	15

CAD = coronary artery disease, FU = follow-up, OMT = optimal medical treatment, SG = stent graft, TEVAR = thoracic endovascular aortic repair, TAAD = type A aortic dissection.

In group B, 5 patients experienced aortic-related adverse events, and which was none in group A (*P* = .045). The saccular aneurysms were noted in 3 patients at 1 month, 6 months, and 12 months during follow-up. Two patients experienced the progression of IHM after 1 and 3 months, respectively. All patients were converted to TEVAR successfully.

**Figure 4.**
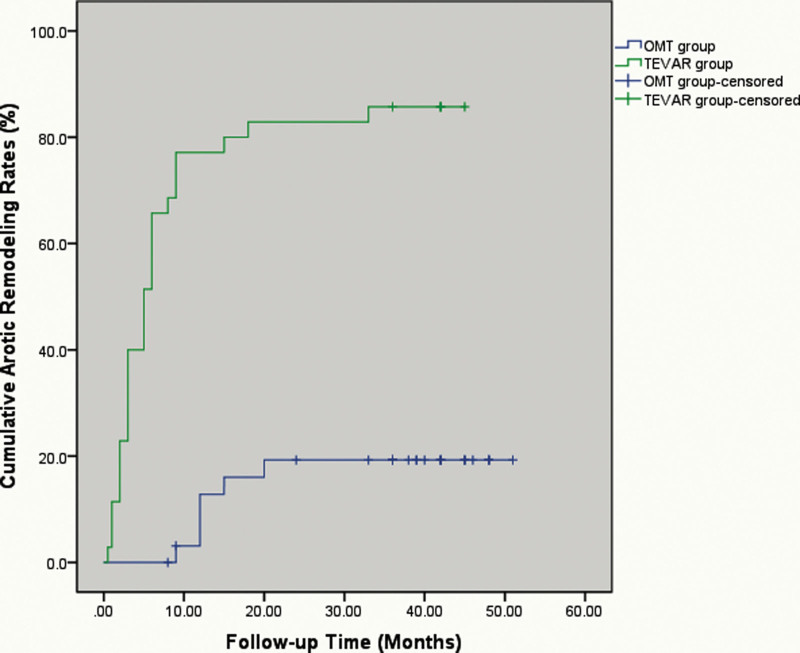
The cumulative aortic remodeling rates.

During follow-up, 18 patients exhibited partial aortic remodeling and 6 patients exhibited complete aortic remodeling in group B. There were 30 patients with complete aortic remodeling and 5 patients with partial aortic remodeling detecting in group A during follow-up (*P* < .001) (Fig. 4). TEVAR-treated patients experienced significantly more complete aortic remodeling than medically treated patients during mid-term follow-up.

## 4. Discussion

Type B acute aortic syndrome is a life-threatening condition including dissection, IMH and PAU. Complicated type B acute aortic syndrome is defined as the presence of organ ischemia, refractory pain, progression of dissection, and rupture.^[6]^ A primary entry tear at the concavity of the distal aortic arch results in a significant increase in the occurrence of complicated type B aortic syndrome.^[7]^ Open surgery for complicated type B aortic syndrome is associated with prohibitive morbidity and mortality, ranging from 2% to 20%.^[8]^ Therefore, TEVAR is an attractive and minimally invasive alternative.^[8–10]^ Early TEVAR for chronic type B dissection with a patent false lumen result in a good prognosis and favorable aortic remodeling.^[11]^ For patients with uncomplicated type B acute aortic syndrome, antihypertensive treatment rather than surgery is acceptable for initial management.^[12]^ However, results from the INSTEAD-XL trial encourage the use of TEVAR in patients with stable type B aortic dissection and suitable anatomy due to optimal long-term benefit.^[10]^

PAUs occur in older patients with severe atherosclerosis subsequent to inflammatory erosion of the intima and result in penetration of aortic blood beyond the true lumen. IMH is a related condition presenting as medial hemorrhage or a microscopic intimal tear.^[13]^ The acute rupture rate of PAU is higher than that of IMH or aortic dissection.^[6,14]^ Complicated PAU is defined as the presence of recurrent pain, an initial diameter >20 mm or a depth >10 mm, or progression of total aortic diameter.^[3]^ According to previous study, the patient with acute type B PAU associated with IMH had a high risk of aortic rupture.^[15]^ Patients with uncomplicated type B PAU are mainly treated by medical treatment and intensive monitoring,^[16]^ while surgical management, including open surgery and TEVAR, is indicated for complicated PAU.^[17,18]^ In the present study, TEVAR was indicated for patients that showed early clinical or radiologic signs of deterioration which was similar to the previously published studies. In group B, 5 patients were transferred to TEVAR due to the progression of PAU (2 patients with the progression of IMH, and 3 patients with the presence of saccular aneurysms). The procedure was performed in the acute phase for 2 patients and in the subacute phase for 3 patients.

Treatment for PAU aims to prevent progression to classic dissection, aortic rupture and promote the aortic remodeling. According to previous study, 35% of the medically treated patients and 55% of TEVAR-treated patients would experience the false lumen thrombosis.^[19]^ For patients with type B PAU undergoing TEVAR, 93% of the patients got complete aortic remodeling according to the report of Liu et al.^[20]^ Ye et al reported that the occurrence of complete aortic remodeling in TEVAR group was significantly higher than in OMT group (82.1% vs 15.4%) for patients with IMH during a mean follow-up of 32 ± 19 months.^[21]^ Close follow-up has been suggested for PAUs, particularly in the case of symptomatic disease, which is more likely to undergo radiographic progression with requirement of intervention.^[22]^ Thirty patients had complete aortic remodeling in TEVAR group, and 6 patients experienced complete aortic remodeling in OMT group during follow-up in our study. Therefore, TEVAR promoted complete aortic remodeling more significantly.

Contemporary studies of TEVAR for type B PAU reported technical success rates ranging from 92% to 100% and in-hospital mortality ranging from 0% to 14.7%.^[5,23–26]^ Some adverse events associated with TEVAR included endoleak, stroke and paralysis.^[5,25]^ Ourania et al reported that 1 of 31 patients with PAU developed paraplegia after TEVAR. Paraplegia following TEVAR seems to be associated with female sex, long segment coverage, and aneurysmal disease.^[27]^ Improved survival was observed with initial surgical management rather than medical management alone.^[14]^ In this study, technically successful deployment of the stent-grafts was performed in all patients in group A. During follow-up, the aortic-related mortality in TEVAR and OMT group was 0% and 13.3%, respectively. All aortic-related death in OMT group resulted from the rupture of aorta. Comparing with patients undergoing TEVAR, OMT treated patients had a significant higher risk of aortic-related mortality. However, patients in OMT group had an insignificant but high risk of all-cause mortality. There was no incidence of aortic-related adverse events in TEVAR group, and there were 5 aortic-related adverse events in OMT group. The TEVAR treated patients had a preferable prognosis in absence of aortic-related death and adverse events during a mean follow-up of 36.13 ± 11.40 months.

PAU usually presents with a preferable proximal landing zone, and endoleak rarely occurred. Furthermore, patients with PAUs are usually older and have several comorbidities leading to a high-risk during surgery.^[6]^ Compared with open surgery, TEVAR seems to be a safe and feasible alternative for patients with type B PAUs.^[3,5,28]^ Perioperative complications occurred rarely due to the minimally invasive nature of the procedure and the utilization of recently developed endovascular devices and techniques. Common complications, such as endoleak and transient spinal ischemia, may occur in elderly patients with several comorbidities.^[5,25,28]^ One patient in TEVAR group had type I endoleak upon immediate post-operative angiography, which disappeared during repeated angiography about 10 minutes later. Re-expansion of the stent-graft made the PAU completely excluded gradually. There was no peri-operative complication required reintervention.

There were several limitations about this study. It was based on a retrospective study rather than a prospective randomized-control trail (RCT). The selection bias should be considered when interpreting the outcomes. Furthermore, the experience of a single-center study with limited number of patients may not be applicable generally, and a multi-center RCT is mandatory to confirm these findings.

## 5. Conclusion

Patients with PAU associated with IMH managed with TEVAR experienced fewer aortic adverse events, lower rate of aortic-related mortality and more incidence of complete aortic remodeling. TEVAR seems to be associated with a better prognosis than OMT treated only.

## Author contributions

All authors help to perform the research. Xiuchun Xu contributed to design, manuscript writing, statistical analysis, critical revision; Fen Lu and Li Li contributed to conception and design, manuscript writing, statistical analysis, critical revision.

**Conceptualization:** Xiuchun Xu, Feng Lu, Li Li.

**Formal analysis:** Xiuchun Xu, Feng Lu, Li Li.

**Funding acquisition:** Xiuchun Xu.

**Writing – original draft:** Xiuchun Xu, Feng Lu, Li Li.

**Writing – review & editing:** Xiuchun Xu, Feng Lu, Li Li.
